# The Impact of Rare Copy Number Variants on Real-World Functional Outcomes in Individuals With Psychosis

**DOI:** 10.1192/bjo.2024.181

**Published:** 2024-08-01

**Authors:** Kimberley Kendall

**Affiliations:** Cardiff University, Cardiff, United Kingdom

## Abstract

**Aims:**

Individuals with psychosis experience impairments in real-world functional outcomes such as employment and health. Rare copy number variants (CNVs) are established risk factors for psychosis, neurodevelopmental disorders and cognitive impairment. However, little is known about their effect on real-world functional outcomes in individuals with psychosis.

I aimed to establish the effect of rare neurodevelopmental CNVs on real-world functioning in individuals with psychosis.

**Methods:**

I identified 1,932 individuals with psychotic disorders (ICD–10 F20–F29) in the UK Biobank using first-occurrence data (from primary care, hospital inpatient and death register records and self-reported conditions). I mapped UK Biobank data to two domains of real-world functional outcomes – health deficits and vocational outcomes. We previously called CNVs using PennCNV, annotating them with 53 CNVs associated with autism spectrum disorder and developmental delay. I conducted regression analyses with neurodevelopmental CNVs as the predictor, real-world functioning as outcomes and with relevant covariates (e.g. age and sex).

**Results:**

Out of 1,932 individuals with psychotic disorders, 2.5% (n = 49) carried a neurodevelopmental CNV.

**Health Deficits**

I used first-occurrence diagnosis data to establish comorbid psychiatric diagnoses. I summed these diagnoses and dichotomised them into one or more comorbid diagnoses versus no comorbid psychiatric diagnoses. I conducted a logistic regression analysis – neurodevelopmental CNV carrier status was associated with having at least one psychiatric diagnosis in addition to a psychosis diagnosis (OR 2.1, 95% CI 1.1 - 4.1, p 0.034). Post-hoc analyses revealed an increased rate of dissociative and conversion disorders in CNV carriers (OR 4.5, 95% CI 1.26 - 15.99, p 0.021).

I used first-occurrence physical health diagnosis data to establish the burden of the 20 most prevalent chronic non-cancer illnesses. Neurodevelopmental CNV carrier status was associated with chronic physical health multimorbidity in individuals with psychosis (59.2% vs 43.5%, OR 2.30, 95% CI 1.27–4.17, p 0.006), defined as the presence of two or more chronic physical health conditions.

**Vocational Outcomes**

I conducted an ordinal regression analysis, establishing that among individuals with psychosis, CNV carriers had a lower likelihood of achieving a higher qualification (OR 0.45, 95% CI 0.27–0.77, p 0.003).

**Conclusion:**

Neurodevelopmental CNVs are associated with important real-world functional outcomes in individuals with psychosis. This work provides information that can guide the assessment and management of individuals with both psychosis and neurodevelopmental CNVs.

